# Payload-Free Epitope Imprinted Polymer Nanoparticles Selectively Targeting IL-13Rα2 and Inhibition of Diffuse Midline Glioma Cells

**DOI:** 10.3390/cancers18183028

**Published:** 2026-09-17

**Authors:** Damla Ulker, Adem Ozleyen, Dmitry Pshezhetskiy, Elena Piletska, Salvador Macip, Nikolai A. Barlev, Sergey Piletsky

**Affiliations:** 1 School of Mathematical and Physical Sciences, University of Sheffield, Sheffield S3 7HF, UK; 2 Department of Pharmaceutical Basic Sciences, Faculty of Pharmacy, Near East University, Near East Bvd, TRNC Mersin 10, 99138 Nicosia, Türkiye; 3 School of Chemistry, University of Leicester, Leicester LE1 7RH, UK; 4 Türkiye Biotechnology Institute, Health Institutes of Türkiye, 06270 Ankara, Türkiye; 5 Norwich Medical School, University of East Anglia, Norwich NR4 7TJ, UK; 6 Mechanisms of Cancer and Aging Laboratory, Department of Molecular and Cell Biology, University of Leicester, Leicester LE1 7RH, UK; 7 Epi4health, Faculty of Health Sciences, Universitat Oberta de Catalunya, 08018 Barcelona, Spain; 8 Department of Biomedical Sciences, School of Medicine, Nazarbayev University, Astana 01000, Kazakhstan

**Keywords:** molecularly imprinted polymers, synthetic antibody nanoparticles, IL-13Rα2, diffuse midline gliomas, glioma cell apoptosis

## Abstract

Background/Objectives: Diffuse midline gliomas (DMGs), including diffuse intrinsic pontine glioma (DIPG), present a significant medical challenge due to their aggressive nature and poor prognosis. Widespread overexpression of the interleukin-13 receptor subunit alpha-2 (IL-13Rα2) across gliomas makes it a promising therapeutic target. Method: Using epitope mapping through a molecular imprinting (EMMI) technique, we developed nanosized molecularly imprinted polymers (nanoMIPs) that specifically recognize different IL-13Rα2 domains. Results: Functional assays showed that nanoMIPs binding to the surface of IL-13Rα2 reduced glioma cell viability, while sparing non-tumor cells. Consistent with receptor-mediated interactions, these nanoMIPs were selectively accumulated onto glioma cells, a specificity validated using a reverse-epitope nanoMIP negative control (nanoMIP-1*). IL-13Rα2-specific nanoMIPs suppressed phosphorylation of ERK1/2, AKT, and STAT3, disrupting major survival pathways. Moreover, these nanoMIPs activated the pro-apoptotic caspase-3/7 pathway, confirming apoptosis induction. Conclusions: Our findings demonstrate that IL-13Rα2-specific nanoMIPs, without a pharmacological payload, exert strong anti-proliferative and pro-apoptotic effects on DMG cells. Collectively, nanoMIPs represent a robust, reproducible synthetic antibody nanoplatform for targeted therapies in cancer and other diseases characterized by cell-surface protein dysregulation.

## Simple Summary

Diffuse midline gliomas are aggressive brain tumors that are particularly difficult to treat because they are located in sensitive areas of the brain and respond poorly to current therapies. A protein called interleukin-1 receptor alpha-2 is highly expressed on many glioma cells but largely absent from healthy cells, making it an attractive treatment target. In this study, we developed nanosized molecularly imprinted polymer particles that can recognize specific regions of the receptor protein. The nanoparticles selectively interacted with patient-derived glioma cells and reduced their survival, while showing highly limited effect on non-tumoral cells. They also blocked important signals that support tumor growth and activated programmed cell death. These findings support the potential of synthetic antibody-like nanoparticles as a new targeted approach for payload-free treatment of aggressive brain tumors.

## 1. Introduction

Diffuse midline gliomas (DMGs), including diffuse intrinsic pontine glioma (DIPG), are aggressive pediatric tumors with extremely poor prognosis. Their midline location prevents surgical resection, and despite radiotherapy and chemotherapy, long-term survival remains under 45% [1,2,3,4]. Recent advances in understanding the molecular profile of DMGs have shifted in treatment strategies toward more specific molecular targeting approaches. A primary therapeutic challenge includes the need for molecularly targeted drugs that can accumulate in the brain at therapeutically effective concentrations by crossing the blood–brain barrier (BBB).

Currently, personalized therapeutic strategies for DMG primarily target dysregulated receptor tyrosine kinases or ion channels such as transient receptor potential melastatin (TRPM2) [5]. Inhibitors of histone-modifying enzymes have also gained attention, particularly for tumors harboring the histone H3-K27M mutation widely present in DMG and DIPG [6,7,8,9,10]. However, histone-modification regulators, VEGF inhibitors and EGFR inhibitors have shown limited efficacy, and several drug candidates, including antibody therapies and immune cell therapies, have been associated with safety concerns in clinical settings [11,12,13,14,15,16].

The interleukin-13 receptor (IL-13R) family, particularly IL-13Rα2, represents a highly relevant target in DMG [13,17,18,19,20,21,22,23,24,25,26,27,28,29,30]. IL-13Rα2 is overexpressed in approximately 80% of high-grade gliomas [6,24] but minimally expressed in normal glia, making it an attractive therapeutic target. This receptor promotes glioma growth by enhancing PI3K/AKT and SRC signaling [31], while its suppression selectively inhibits glioblastoma cell proliferation [32]. Similarly, antibody targeting of IL-13Rα2 potently inhibits activation of FAK, SRC, and PI3K/AKT pathways, significantly reducing cancer cell proliferation [33].

Previous attempts to target IL-13Rα2, including monoclonal antibodies, immunotoxins, and peptide-based systems, showed promise in preclinical models but failed to achieve consistent efficacy in clinical trials due to poor BBB penetration [9,17,21,27,29,34], limited tolerability of the dosing regimen, and off-target effects [13,14,19,28,35,36]. More recently, short IL-13Rα2-targeting cell-penetrating peptides fused to membrane-degrading peptides or peptide-hybrid nanoparticulate systems bearing toxic payloads have been developed, probing the suitability of nanoparticle-based anti-glioma therapies [37,38].

In this regard, nanoMIPs offer a promising strategy for designing IL-13Rα2 inhibitors given their unique molecular recognition properties. Numerous examples of successful nanoMIPs implementation against various biological molecules confirm their selectivity and strong binding affinities to specific targets in both cellulo and in vivo [39,40]. Several studies have explored nanoMIPs as drug delivery vehicles for anticancer therapies, highlighting their potential for targeted and controlled release of chemotherapeutics [41,42,43,44,45,46]. Importantly, while previous formulations developed in our laboratory have demonstrated the capacity to cross the BBB in vivo [47], this current work establishes the foundational in vitro therapeutic efficacy and signaling pathway disruption of target-specific variants directly within patient-derived DIPG cells.

A significant advancement in nanoMIPs-based nanomedicine was the advent of EMMI (epitope mapping by molecular imprinting), which enables rapid identification of suitable epitopes for targeted nanoMIP fabrication against extracellular receptors, proteins, or whole cells [48,49,50]. Building on these advancements, we have adapted the nanoMIP-based EMMI approach to identify and select the most active nanoMIPs against the IL-13Rα2. Among the synthesized nanoMIPs, several were able not only to target the IL-13Rα2-expressing cancer cells but also to internalize into tumor cells and subsequently induce apoptosis. These results provide a strong rationale for the use of targeted nanoMIPs for therapeutic purposes, either as single therapeutics or as adjuvants with other drugs.

## 2. Experimental Section

### 2.1. Materials

All chemical reagents were obtained Sigma Aldrich (St. Louis, MO, USA) unless stated otherwise: glass beads (150 µm), anhydrous toluene, *N*-isopropylacrylamide (NIPAm), *N*-tert-butylacrylamide (TBAm), *N*-(3-aminopropyl)-methacrylamide hydrochloride (APMA), *N-N′*-methylene-bis-acrylamide (MBAA), acrylic acid (AA), fluorescein *O*-methacrylate (FOMA), *N,N,N′,N′*-tetra methyl ethylenediamine (TEMED), potassium persulfate (KPS), trypsin (from bovine pancreas), 1-ethyl-3-(3-dimethylaminopropyl) carbodiimide hydrochloride (EDC), N-hydroxysuccinimide (NHS). Phosphate-buffered saline (PBS) tablets, potassium chloride, sodium chloride, ethanol and ethanolamine (99%) were obtained from Thermo Scientific (Waltham, MA, USA). Recombinant interleukin-13 receptor subunit alpha-2 (IL-13Rα2) was purchased from AcroBiosystems (Newark, DE, USA). Octet^®^ amine-reactive 2nd generation (AR2G) biosensor kits and 96-well, black, flat-bottom, polypropylene microplates were obtained from Sartorius (Göttingen, Germany) and Greiner Bio-One (Kremsmünster, Austria).

HSJD-DIPG-007 patient-derived in vitro primary cell cultures were established by Angel Montero Carcaboso at Hospital San Joan de Déu (Barcelona, Spain) and SU-DIPG-36 by Michelle Monje at Stanford University (Stanford, CA, USA) [51]. The immortal human–human hybrid cell line MO3.13 that expresses phenotypic characteristics of primary oligodendrocytes was obtained from Cedarlane Labs (CLU301, Burlington, ON, Canada) and maintained in high-glucose DMEM (D5796) plus 10% FBS (Gibco, Frederick, MD, USA, A5670801).

Cell culture reagents were purchased from Intogen, Beverly, MA, USA: Neurobasal-A medium (1X), D-MEM/F-12 (1X), liquid, 1:1, HEPES buffer solution (1M), MEM sodium pyruvate solution (100 mM) (100X), MEM non-essential amino acids solution 10 mM (100X), GlutaMAX-I supplement, and B-27 supplement minus vitamin A (50X). Recombinant human proteins (H-EGF, H-FGF-basic-154, H-PDGF-AA, H-PDGF-BB) were purchased from Shenandoah Biotech (Warminster, PA, USA). Heparin solution (0.2%) was obtained from StemCell Technologies, Inc. (Vancouver, BC, Canada). Cultrex Laminin 1 was purchased from Bio-Techne (Minneapolis, MN, USA); D-PBS (with Calcium and Magnesium), and Accutase were from Sigma-Aldrich. RIPA lysis buffer was obtained from Santa Cruz Biotechnology (Dallas, TX, USA). STEM-CELLBANKER was from AMS Biotechnologies (Cambridge, UK). CellBrite^®^ Steady 650 nm far-red fluorescent membrane dye was purchased from Biotium (Fremont, CA, USA).

Primary antibodies were purchased from Cell Signaling Technology (Danvers, MA, USA): p44/42 MAPK (ERK1/2; 44 kDa), diphosphorylated ERK1/2 (42 kDa), AKT (60 kDa), phospho-AKT (Ser473-60 kDa), STAT3 (86 kDa), and phospho-STAT3 (Tyr705-88 kDa). Anti-GAPDH (36 kDa) antibody was purchased from Abcam (Cambridge, UK). Goat anti-rabbit IgG and goat anti-mouse IgG secondary antibodies were obtained from LI-COR IRDye^®^ (Lincoln, NE, USA). Coomassie dye reagent, Bis-Tris gels (4–12%), Eppendorf™ Safe-Lock Tubes 1.5 mL, Snakeskin dialysis tubing (10K MWCO, 22 mm) and T25/T75 cell culture flasks were purchased from Thermo Scientific. CellTiter-Glo^®^ Viability Assay, Caspase-Glo^®^ 3/-7 Assay, and pan-caspase inhibitor Z-VAD-FMK were purchased from Promega (Madison, WI, USA). Mycoplasma detection kits (MycoStrip™) were purchased from InvivoGen (San Diego, CA, USA). Polymer coverslip chambers for confocal imaging were purchased from Ibidi (Gräfelfing, Germany).

### 2.2. Identification of Specific Epitopes Using Molecular Imprinting

The epitopes were identified using a well-established mapping method for IL-13Rα2. Briefly, a monomer mixture was prepared by dissolving 19.5 mg of NIPAm, 15 mg of TBAm (dissolved in 200 µL of ethanol), 3 mg of APMA, 3 mg of MBAA, and 50 µL of acrylic acid solution (22 µL of acrylic acid in 1 mL H_2_O) in 50 mL of PBS. The solution was purged with nitrogen gas for 20 min. Afterwards, 200 µg of IL-13Rα2 protein was added to 50 mL of degassed monomer mixture. Polymerization was initiated by adding TEMED and freshly prepared KPS (6 µL of TEMED, 12 mg KPS in 400 µL of PBS). The polymerization was conducted for 1 h at room temperature.

Following polymerization, unreacted monomers and low-affinity materials were removed by washing with 4 × 5 mL of PBS using a centrifugal filter for 12 min at 2355 *g* (3500 rpm). Next, 0.8 mg of trypsin in 8 mL of PBS was added to the centrifuge cartridge containing the IL-13Rα2-immobilized MIPs and incubated at room temperature for 72 h. After incubation, free fragments of digested proteins and trypsin were removed by centrifugation using a 50 kDa centrifugal filter for 10 min at 2355 *g* using 4 × 10 mL of HPLC-grade water. In the final step, 1 mL of hot water (95 °C) was added to the peptide-MIP solution remaining inside the cartridge. This solution was transferred to a 1.5 mL Eppendorf tube and incubated at 95 °C for 3 min. Centrifugation was repeated three times under the same conditions to fully dissociate and collect the template peptides from MIP cavities. The collected solutions were pooled into a 15 mL tube for the liquid chromatography-mass spectrometry (LC-MS) sequence analysis. Lou et al. introduced high-performance liquid chromatography-tandem mass spectrometry for the plasma sample identification; however, its suitability for our method limits its utility in this context [52]. Details of full LC-MS analysis are provided in the supporting information.

### 2.3. Design and Synthesis of the NanoMIPs Using Solid-Phase Polymerization

The nanoMIPs were engineered with recognition sites designed to selectively bind specific target peptide domains on the IL-13Rα2 extracellular receptor. Glass beads (90 µm, 60 mg) were used as the solid support matrix. To activate the surface, the glass beads were boiled in hot ethanol (70 °C for 30 min) and dried in the oven at 70 °C for 3 h. The hydroxide activated glass beads were then incubated with (3-iodopropyl) trimethoxysilane in 25 mL of dry toluene for silanization overnight at room temperature. Following the reaction, the glass beads were washed with 5 × 50 mL of acetone and 3 × 30 mL of ethanol and dried at 80 °C for 30 min. The silanized glass beads were transferred to a light-protected flask, where 10 mg of customized, cysteine-capped epitope peptide dissolved in 20 mL of 30 mM borate buffer was added. Following overnight incubation, 8 µL of mercaptoethanol was added to quench unreacted silane groups, followed by 30 min of incubation. The glass beads were then washed five times with 100 mL of water and six times with 50 mL of acetone.

Ten different nanoMIPs formulations were synthesized through solid-phase polymerization driven by self-assembly interactions around the template epitopes. The polymerization mixture contained 0.34 mmol NIPAM, 0.24 mmol TBAm, 0.034 mmol APMA, 0.31 µmol AA, and 2.50 µmol FOMA (for fluorescent tracking), and 0.31 mmol MBAA cross-linker. Polymerization was initiated in PBS at room temperature for 1 h using 0.55 mmol KPS and 0.045 µmol TEMED. Because traditional non-imprinted polymers (NIPs) do not replicate the surface chemistry or synthesis kinetics of solid-phase peptide-templated polymerization, a reverse epitope sequence (nanoMIP-1*) was synthesized using identical protocol to serve as rigorous negative control for non-specific material-cell interactions.

Following polymerization, unreacted monomers and low-affinity polymer fractions were removed by cold water washes (5 × 20 mL). High-affinity nanoMIPs were subsequently isolated using a hot ethanol wash 5 × 30 mL (70 °C). The collected nanoMIP solutions were concentrated using a rotary evaporator and nanoMIPs underwent dialysis for five days against water. Purified nanoMIPs were freeze-dried overnight for further characterization.

### 2.4. Characterization

The z-average hydrodynamic diameter and size distribution (polydispersity index, PDI) of the nanoMIPs were determined using a Malvern Zetasizer NanoZS (Worcestershire, UK) instrument at a fixed scattering angle of 173°. Samples were diluted to 0.1% *w*/*w* using either deionized water at room temperature or evaluated after 24 h incubation in PBS and TMS cell media at 37 °C. Data were averaged over three consecutive measurements (using ten sub-runs per run). The hydrodynamic diameter was calculated using the Stokes–Einstein equation.

For structural morphology assessment via Transmission Electron Microscopy (TEM), continuous carbon-coated copper grids (200 mesh, 3 mm diameter; Agar Scientific, Rotherham, UK) were plasma glow-discharged for 30 s to create a hydrophilic surface. An 8 µL droplet of a 0.03% *w*/*w* aqueous nanoMIPs dispersion was placed on a surface-treated grid for 1 min, and excess solution was removed with filter paper. Negative staining was performed by applying an 8 µL droplet of a 0.75% *w*/*v* aqueous solution of uranyl formate (Electron Microscopy Science, Hatfield, PA, USA) for 30 s. Grids were dried using a vacuum hose and imaged on a JEOL 2100 TEM instrument at 120 kV equipped with an EMSIS Xarosa 20-megapixel digital camera with Radius software, 2019.

To evaluate target binding affinity kinetics, bio-layer interferometry (BLI) was performed using a Sartorius Octet QKE system. Amine-reactive 2nd generation (AR2G) biosensors were hydrated in water for 5 min and washed with 1X kinetic buffer for 5 min. Biosensor carboxylic acid groups were activated using 400 mM EDC and 200 mM NHS for 5 min to form NHS esters. Amine coupling was achieved by incubating the activated biosensors in a 100 ppm solution of each nanoMIP variant dissolved in kinetic buffer for 30 min. Unreacted esters were quenched with 1 M ethanolamine (pH 8.5) followed by a 10 min wash in kinetic buffer. Following a 5 min baseline setup, the nanoMIPs-functionalized biosensors were immersed in a 10 ppm solution of recombinant IL-13Rα2 protein in 10 mM acetate buffer (pH 4). Initial screening identified nanoMIP-1, nanoMIP-3 and nanoMIP-8 as lead candidates. Detailed dissociation constant (K_d_) determinations were subsequently performed using a range of IL-13Rα2 protein concentrations (from 100 ppm down to 0.625 ppm) in 10 mM acetate buffer (pH 4). Association and dissociation phases were recorded for 30 min and 10 min, respectively, and data were fitted using a 1:1 global binding method.

### 2.5. Cell-Based Activity Screening of NanoMIPs

Patient-derived pediatric glioma cell lines HSJD-DIPG-007 and SU-DIPG-36, along with the non-tumoral immortalized human glial cell line MO3.13 (serving as a targeting control), were provided by Prof. Chris Jones (Division of Molecular Pathology and Glioma Group). HSJD-DIPG-007 and SU-DIPG-36 cells were cultured in Tumor Stem Medium (TSM) consisting of a 1:1 (*v*/*v*) mixture of Neurobasal-A medium (1X) and DMEM/F-12 (1X). TSM was supplemented with HEPES buffer solution, MEM sodium pyruvate solution, MEM non-essential amino acids, and GlutaMAX-I, B-27 supplement, human EGF (20 ng/mL), human FGF-basic-154 (20 ng/mL), human PDGF-AA (10 ng/mL), human PDGF-BB (10 ng/mL), and heparin (2 µg/mL). MO3.13 cells were maintained in high-glucose DMEM supplemented with 10% (*v*/*v*) fetal bovine serum (FBS). All cell lines were maintained at 37 °C in a humidified incubator with 5% CO_2_.

For glioma stem cell lines, culture flasks were pre-coated with Cultrex Laminin (2.66 µg/mL in DPBS containing Ca^2+^ and Mg^2+^) by incubating at 37 °C for 2 h. Excess laminin was removed, and flasks were rinsed with fresh media before cell seeding. Cells were incubated in these prepared flasks, and cell media was refreshed every 48 h, and cells were detached at 70–80% confluence using Accutase for 2 min at 37 °C. Cells were verified to be negative for mycoplasma contamination every three passages using the MycoStrip^TM^ kit. NanoMIP treatments were initiated after overnight attachment at seeding densities of 1000 cells/well for HSJD-DIPPG-007 and 500 cells/well for SU-DIPG-36 and MO3.13 in 96-well plates.

### 2.6. Preparation of NanoMIPs for Cell Treatments

NanoMIP stability was monitored across various media profiles using DLS. Hydrodynamic diameters demonstrated excellent stability when transforming from aqueous media at 25 °C to ionic PBS and TMS frameworks at 37 °C. Working stock formulations were prepared by dissolving purified freeze-dried nanoMIPs in sterile PBS at 1 mg/mL and stored at 4 °C. Prior to cell treatment, stocks were diluted to 100 ppm and sterilized under UV light for 15 min and briefly homogenized via sonication (30 s, 2 cycles, 50/60 Hz, Diagenode SA sonicator, Liège, Belgium). Sterile vehicle-only PBS solutions served as treatment controls.

### 2.7. Cell Titer-Glo (CTG) Viability Assay

Cells were seeded into laminin-coated 96-well plates at densities of 1 × 10^4^ cells/well (HSJD-DIPG-007) or 0.5 × 10^3^ cells/well (SU-DIPG-36) and allowed to adhere overnight. Cells were subsequently treated with 100 ppm of each of the ten individual nanoMIP variants for 24, 48 and 72 h. The non-tumoral MO3.13 cell line (1 × 10^4^ cells/well) was evaluated in parallel against lead candidates to determine cell-type toxicity profiles. This nanoMIP concentration was modified from one of our previous cell receptor targeting studies [45]. The reverse sequence formulation (nanoMIP-1*) was evaluated across all lines to serve as material-specific negative control. Assays were performed using three technical replicates. At the designed endpoint, CellTiter-Glo^®^ luminescent reagent was added directly to the wells, and luminescence corresponding to metabolic intracellular ATP concentration was recorded on a Hidex Sense plate reader (LabLogic, Sheffield, UK). Viability data were normalized against vehicle-treated control values.

### 2.8. Cellular Interactions of nanoMIPs

Real-time cellular targeting and localization kinetics were evaluated using Nikon C1Si confocal laser scanning microscopy (Tokyo, Japan). Cells were seeded into Ibidi polymer coverslip chambers at densities of 2 × 10^4^ (HSJD-DIPG-007) or 1 × 10^4^ and 1 × 10^4^ (SU-DIPG-36 and MO3.13) cells/well, respectively, and incubated for 24 h. Prior to live imaging, cell membranes were stained using CellBrite^®^ Steady 650 nm far-red fluorescent membrane dye for 30 min. Following membrane staining, 100 ppm of fluorescently labeled (green-520 nm) nanoMIPs were added directly to the chamber wells. Live-cell confocal imaging was performed on a Nikon C1Si microscope (Tokyo, Japan) equipped with an automated incubation enclosure. Intermittent captures were compiled every 10 s over a continuous 30 min window. Images were collected using fixed filter tracks (red channel: 900–2500 nm; green channel: 500–4000 nm) at an acquisition speed of 15 fps. Quantified mean fluorescence tracking and cross-sectional spatial distribution analysis were generated using Imaris Image Analysis software, 2023.

### 2.9. Protein Expression Analyses

Western blot assays were conducted to track downstream signal pathway alterations. Cells were seeded into 6-well plates (2 × 10^6^ cells/well for HSJD-DIPG-007; 1 × 10^6^ cells/well for SU-DIPG-36) and treated with 100 ppm of selected nanoMIP formulations for 30 min and 6 h. Cells were harvested using accutase, collected into tubes, and centrifuged at 1300 rpm for 5 min, followed by three cold PBS washes. Pellets were lysed in ice-cold RIPA buffer supplemented with 1:100 *v*/*v* protease/phosphatase inhibitor cocktails for 20 min incubation on ice. Lysates were cleared by centrifugation at 13,000 *g* for 15 min at 4 °C, and supernatants were collected.

Protein quantification was performed using a standard Bradford assay. Absorbance at 595 nm was recorded on a Hidex Sense Microplate Reader (LabLogic, Sheffield, UK) and interpolated against a BSA standard curve (0–8 µg/µL). Equal protein masses were loaded on 4–12% Bis-Tris and electrophoresed in MES running buffer at 120 V for 80 min, followed by transfer into nitrocellulose membranes using a semi-dry blotting system (Pierce™) at 10 W for 30 min. Membranes were blocked with 5% *w*/*v* BSA in 1X PBST (0.1% Tween-20) for 1 h and incubated with primary antibodies for 3 h at room temperature. Target proteins included total p44/42 MAPK (ERK1/2; 44 kDa), diphosphorylated p-ERK1/2, total AKT (60 kDa), p-AKT (Ser473; 60 kDa), total STAT3 (86 kDa), p-STAT3 (Tyr705; 88 kDa) and GAPDH (36 kDa). Following PBST washing, membranes were incubated with secondary antibodies, including goat anti-rabbit IgG and goat anti-mouse IgG, both diluted 1:10,000, and scanned on an Odyssey CLx infrared imaging system (LI-COR). Band intensities were quantified using Image Studio Lite Ver 2.5 and normalized directly against respective internal GAPDH controls and relative changes compared to the control group. Western blot assays were performed two to three times using three technical replicates for each treatment.

### 2.10. Caspase Activity Assay

Apoptotic activation kinetics were monitored using the luminescent Caspase-Glo^®^ 3/-7 Assay (Madison, WI, USA). Cells (HSJD-DIPG-007; 1000 cells/well and SU-DIPG-36; 500 cells/well) were seeded onto laminin-coated 96-well plates and allowed to adhere overnight. Cells were then treated with 100 ppm of nanoMIP-4, nanoMIP-6, nanoMIP-8 and nanoMIP-1* for 24 h. Cisplatin treatment (25 µM) was implemented as a positive control for caspase-3/7 activation. To verify caspase dependency, parallel experimental tracks were pre-incubated with the pan-caspase inhibitor Z-VAD-FMK (50 µM) for 2 h prior to nanoparticle or cisplatin exposure. Following the 24 h treatment window, 50 µL of Caspase-Glo^®^ 3/7 reagent was added directly to each well and incubated at room temperature for 1 h. Luminescent output was quantified using a Hidex Sense plate reader (LabLogic, Sheffield, UK).

### 2.11. Statistical Analysis

Data gathered from at least three independent biological and/or technical replicates are presented as the mean ± standard error of the mean (SEM). Multi-group variance coordinated were calculated via one-way analysis of variance (ANOVA) followed by Dunnett’s post hoc test generated in GraphPad Prism 9 software boundaries (* *p* < 0.05, ** *p* < 0.005, *** *p* < 0.001). Binary variant tracks were analyzed using unpaired Student’s *t*-tests.

## 3. Results and Discussions

### 3.1. Identification of Functional IL-13Rα2 Epitopes

**Figure 1:**
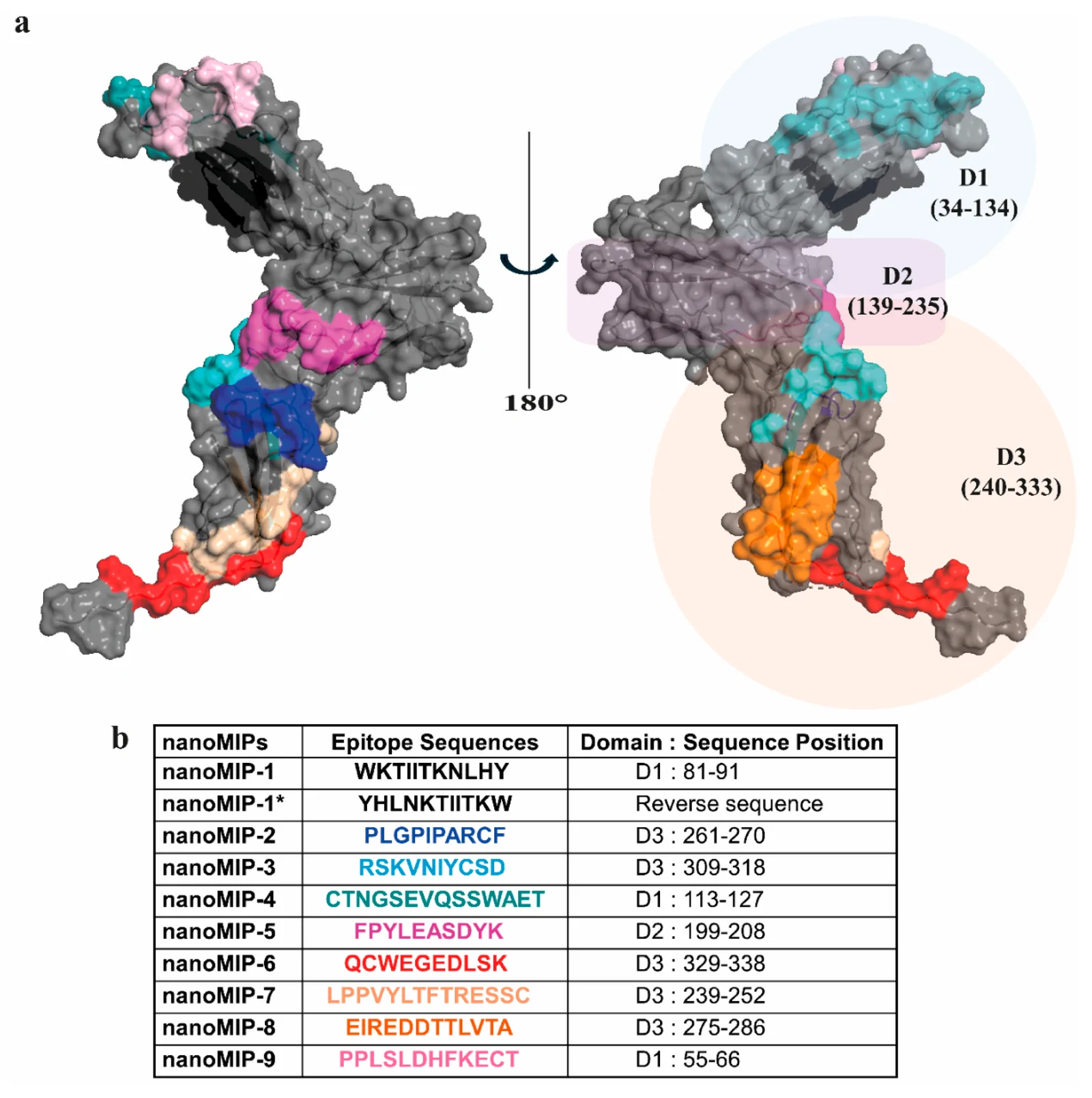
(**a**) Structural locations of epitopes od D1, D2, D3 domains mapped on IL-13Rα2 receptor protein using (PDB: 3LB6) and UniProt (Q14627) 3D model (**b**) selected epitopes from EMMI identification and IL-13/IL-13Rα2 complex interactions to synthesis nanoMIPs to target IL-13Rα2. * The reverse sequence of the first epitope sequence was used as a material control.

To map targeted binding domains across the 3D structure of the IL-13Rα2 receptor, we combined standard structural modeling matching the published IL-13/IL-13Rα2 co-crystal configuration (PDB ID: 3LB6) with our epitope mapping EMMI method [48,50,53,54]. Structural modeling confirmed that primary domains D1 and D3 exhibit high functional importance during ligand binding events (Figure S1). Binding alignments were mapped to site III of IL-13, which corresponds directly to the S-type immunoglobulin fold within the D1 motif of IL-13Rα2 (residues: WKTIITKN), and site II of IL-13, aligning with the fibronectin type III fold inside the D3 motif (residues: PLGPIPARCF). Minor secondary interface properties were also noted along the intermediate D2–D3 boundary region (residues: IYCSD) [17,21,30,35]. The locations of these identified sequence targets were mapped onto the spatial crystal structure of the receptor and are illustrated in Figure 1a. Additionally, targeted epitopes of the receptors were summarized in Figure 1b; the first three epitopes were identified from the IL-13/IL-13Rα2 complex, and the remaining six from protein mapping. All epitopes were cysteine-capped for further functionalization onto the glass bead surface. Structural models were adapted from (PDB: 3LB6) and UniProt (Q14627) using PyMOL 2.4.1.

To complete unbiased whole-receptor extraction using the EMMI method, nanoMIPs were generated against the full intact extracellular domain of the recombinant protein and subsequently exposed to controlled trypsin digestion. Briefly, nanoMIPs were fabricated against the whole IL-13Rα2 protein and digested with trypsin. The cocktail of epitopes protected from trypsin digestion by nanoMIPs was subsequently analyzed by LC-MS, identifying 40 sequences (Table S1). These sequences were filtered based on local abundance coordinates, electron density scores, surface accessibility, evolutionary conservation metrics, and immediate proximity to the active ligand-binding pocket to yield six prioritized operational epitope options. Specifically, sequences extracted from domain D1 (epitopes 4 and 6) and domain D3 (epitope 8) were chosen due to their roles in stabilizing and signaling the IL-13/IL-13Rα2 complex interfaces. Ultimately, nanoMIP1- through nanoMIP-3 were synthesized against the predefined from IL-13/IL-13Rα2 crystal-structure domains, nanoMIP-4 through nanoMIP-9 were built against the EMMI mass spectrometry-derived sequences, and nanoMIP-1* was generated against the reversed primary sequence of Epitope 1 to establish a material (Figure 1a).

### 3.2. The nanoMIPs Synthesis Using Solid-Phase Polymerization

Epitope-specific nanoMIPs were synthesized using a solid-phase polymerization approach where cysteine-capped peptides were covalent linked to silanized glass bead supports [41,53,54,55,56]. This framework allows monomer configuration assembly and cross-linking to occur directly around the anchored peptide templates. Fluorescein *O*-methacrylate was incorporated into the polymerization for cellular interaction studies as described previously [57]. The synthesis scheme has been summarized in Figure S2. Following thermal elution and clean-up cycles, template chains were removed to leave behind structurally stable, imprinted nanocavities that match the spatial conformation of the target epitopes.

DLS analysis confirmed that the synthesized nanoMIPs exhibited a consistent average hydrodynamic diameter of approximately 150 ± 10 nm under ambient processing conditions. To assess stability across physiological testing conditions, DLS size assessments were performed across water, PBS and TMS cell culture media at 37 °C. The nanoMIPs maintained consistent average dimensions of approximately 150 nm ± 20 nm with narrow polydispersity values (PDI ≤ 0.3), indicating excellent physical stability and absence of aggregation in an ionic environment (Table S2). Morphological characterization of freeze-dried nanoMIPs was conducted using TEM, and images indicated uniformly spherical particles, approximately 25 nm in diameter. Representative images were given in Figure S3a–c for selected nanoMIPs. This difference between dry TEM measurements and hydration-state DLS diameters is typical for highly cross-linked polymeric nanonetworks that expand via solvent absorption upon rehydration.

### 3.3. Binding Affinities of nanoMIPs on the IL-13Rα2 Receptor Protein

Real-time binding kinetics between the ten nanoMIP variants and the IL-13Rα2 extracellular target domain were tracked using bio-layer interferometry (BLI). Initial screening assays showed that the recombinant receptor protein possessed high binding affinities for nanoMIP1, nanoMIP-3 and nanoMIP-8, whereases limited binding profiles were observed for the remaining formulations, including the reverse-sequence control nanoMIP-1* (Figure S4a).

Full multi-concentration kinetic analysis was conducted on the lead candidates to determine their dissociation constants (K_d_). The calculated values were 2 × 10^−10^ M (220 pM) for nanoMIP-1, 4.5 × 10^−10^ M (450 pM) for nanoMIP-3, and 5.7 × 10^−10^ M (570 pm) for nanoMIP-8 (R^2^ ≥ 0.99). Notably, the nanomolar binding affinity achieved by nanoMIPs matches or exceeds the natural binding properties reported for the native IL-13/IL-13Rα2 complex (K_d_ = 0.5–1.2 nM) [21].

Interestingly, while nanoMIP-3 demonstrated high physical binding affinity during cell-free BLI analysis, it produced minimal biological cytotoxicity during live-cell screenings. On the other hand, nanoMIP-4, nanoMIP-6 and nanoMIP-8 significantly reduced glioma cell viability; nanoMIP-8 exhibited the highest in vitro binding affinity to IL-13Rα2, with a dissociation constant (K_d_ of 0.57 nM) among ten nanoMIPs. This highlights that raw binding affinity alone is not sufficient to guarantee functional receptor antagonism. Instead, precise epitope placement and matching target domain specificity are the critical drivers of functional activity; nanoMIP configurations designed to interact directly with the core D1, and D3 ligand-binding faces were the variants capable of triggering downstream pathway disruption. This difference underscores the impact of the complex cellular environment, including subcellular locations, surface receptor presentation, structural steric hindrances, glycan masking, and membrane dynamics compared to isolated cell-free protein assay setups [58,59].

### 3.4. The nanoMIPs Effect on Glioma Cells and Non-Tumoral Cell Lines

**Figure 2:**
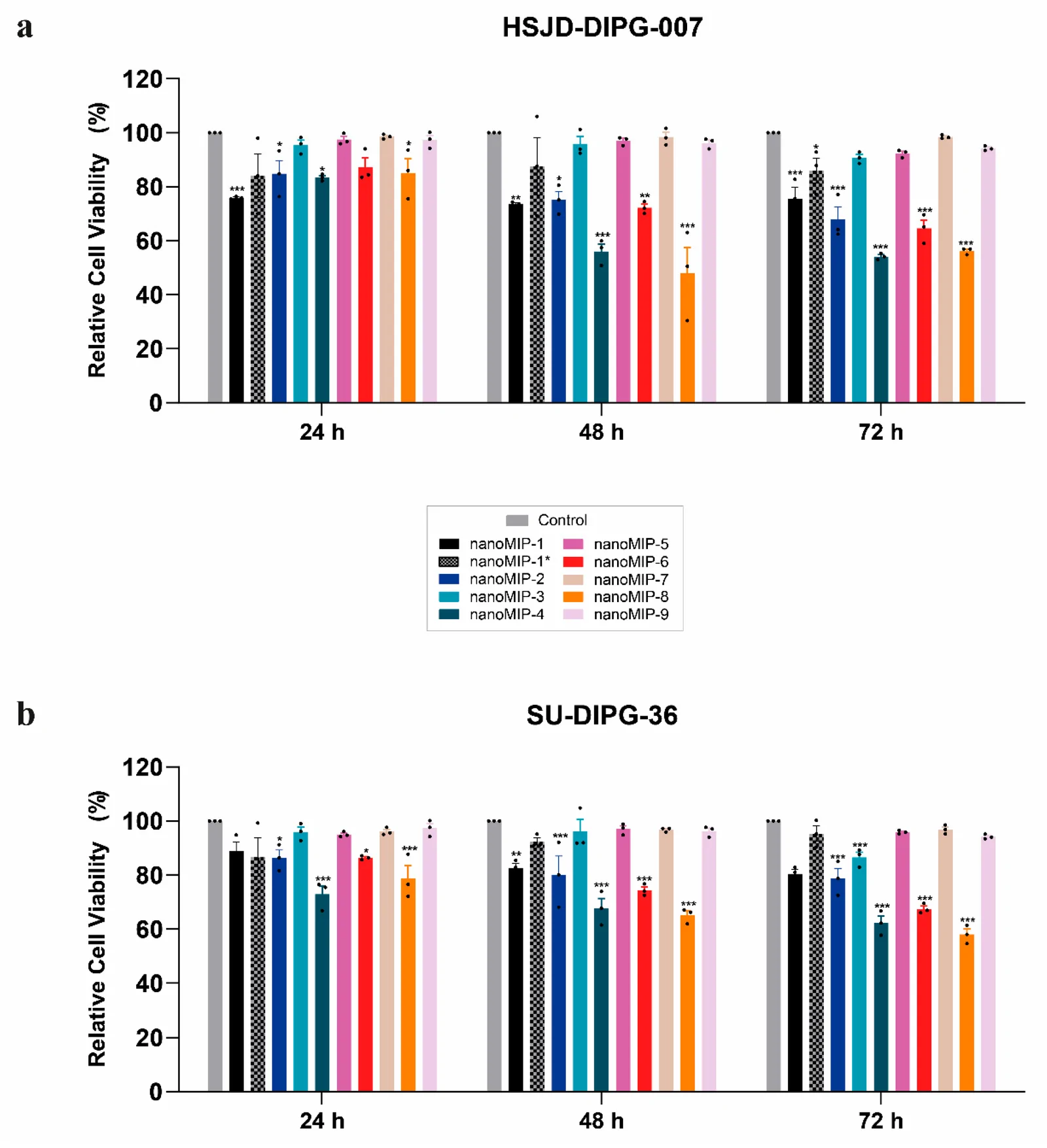
Cell viability profiles of (**a**) HSJD-DIPG-007 and (**b**) SU-DIPG-36 cells treated with the nanoMIPs panel over 24, 48 and 72 h. The data were obtained from at least three technical and/or biological replicates using a Hidex Sense plate reader (LabLogic, Sheffield, UK) to determine the luminescence measurements and are presented as the mean ± standard error of the mean (SEM). Statistical significance between control (C: TMS cell media control) and treatment groups was assessed using one-way analysis of variance (ANOVA) followed by Dunnett’s post hoc test, performed with GraphPad Prism 9 software (* *p* < 0.05, ** *p* < 0.005, *** *p* < 0.001).

To test the in vitro IL-13Rα2 targeting and therapeutic efficacy of the nanoMIP formulations, patient-derived DIPG (HSJD-DIPG-007 and SU-DIPG-36) along with non-tumoral glial control cells (MO3.13) were used as a control [6,7,60,61]. Cell mutations for these three cell lines have been summarized in Table S3. HSJD-DIPG-007, SU-DIPG-36 and MO3.13 cells were treated with 100 ppm of each variant over 24, 48, and 72 h periods. Cell survival tracking showed that nanoMIP-2, nanoMIP-4, nanoMIP-6, and nanoMIP-8 induced significant, time-dependent decreases in metabolic viability across both patient-derived tumor lines. After 72 h of exposure, these lead three nanoMIP-4, nanoMIP-6, and nanoMIP-8 caused a consistent 40–50% reduction in glioma cell viability, whereas nanoMIP-2 cause approximately 30% reduction (Figure 2a,b).

Crucially, parallel evaluations using the non-tumoral human glial MO3.13 line showed no significant cytotoxic response or decrease in viability even after extended exposure to these lead nanoMIP-2, nanoMIP-4, nanoMIP-6, and nanoMIP-8 (Figure S5). Furthermore, the reverse-sequence configuration (nanoMIP-1*) failed to induce toxicity across any of the evaluated cell systems, confirming that the anti-proliferative activity is driven by sequence-specific epitope matching rather than non-specific nanoMIP toxicity. This clear safety margin correlates directly with the selective overexpression of IL-13Rα2 on the surface of aggressive gliomas compared to its near-total absence on healthy glial cell structures.

### 3.5. Cellular Interaction Capacity of nanoMIPs

**Figure 3:**
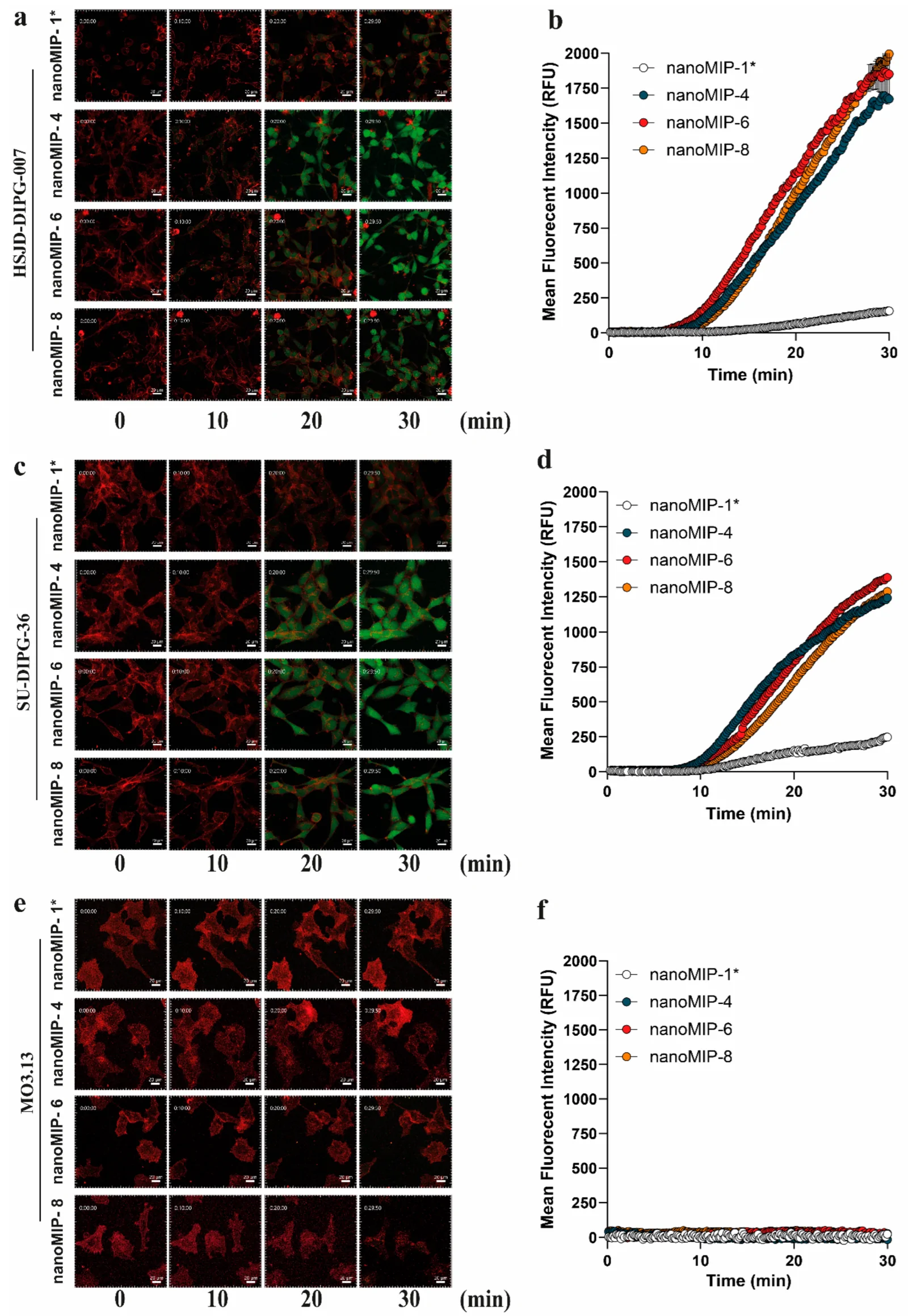
Real-time confocal laser scanning microscopy tracking and mean fluorescent intensity curves of nanoMIP-4, nanoMIP-6, nanoMIP-8 and nanoMIP-1* (reverse sequence) on monolayer cultures of (**a**) HSJD-DIPG-007, (**c**) SU-DIPG-36, and (**e**) MO3.13, respectively. The mean fluorescent intensity of nanoMIP localization on (**b**) HSJD-DIPG-007, (**d**) SU-DIPG-36, and (**f**) MO3.13, respectively. Cells were stained with a far-red fluorescent membrane dye (650 nm, red) for 30 min, then treated with 100 ppm FOMA-labeled nanoMIPs (520 nm, green). Imaging was performed using a Nikon C1Si microscope at 10 s intervals over 30 min. Scale bars represent 20 µm. Mean fluorescent intensity and standard deviation were plotted from cell surfaces and the surrounding areas.

To monitor real-time targeting dynamics, live cells with stained fluorescent membranes were exposed to FOMA-labeled nanoMIP variants and tracked via confocal laser scanning microscopy. Representative images from three independent experiments were given at 0, 10 min, 20 min, and 29.50 min time points (Figure 3a–d). Mean fluorescent intensity curves of nanoMIPs localization on glioma cells or non-tumoral cell members were displayed in Figure 3b–f.

Confocal imaging showed that the functional variants nanoMIP-4, nanoMIP-6 and nanoMIP-8 rapidly localized and accumulated on the membrane of HSJD-DIPG-007 and SU-DIPG-36 cells within 10 min of addition, showing steady intensity increases over the 30 min tracking window. Conversely, the negative control nanoMIP-1* showed no detectable accumulation or bonding on the tumor cells.

When evaluated against the IL-13Rα2-negative non-tumoral MO3.13 cell, the lead three nanoMIPs demonstrated membrane accumulation or interaction, maintaining a flat baseline means intensity curve over time (Figure 3f). The total lack of interaction observed with the reverse control nanoMIP-1*, combined with lack of binding on non-tumoral cells, confirms that these synthetic antibody nanoMIPs achieve highly selective, target-dependent tracking governed exclusively by localized receptor expression profiles.

### 3.6. Mechanism of Action of Targeted nanoMIPs

It is known that three main treatment strategies have emerged in IL-13Rα2 targeted therapy: (1) fully blocking IL-13 binding to IL-13Rα2, (2) targeting the D1 motif (GSETWKIITKN), and (3) targeting the D3 motif (LSLDHKECTVEYEL), which lies very close to the IL-13 binding domain. These approaches aim to block critical active motifs on IL-13Rα2 to inhibit activity and thereby modulate key downstream signaling pathways, including JAK/STAT, PI3K/AKT, Src/ERK, and STAT3/STAT6 [17,25,29,35].

**Figure 4:**
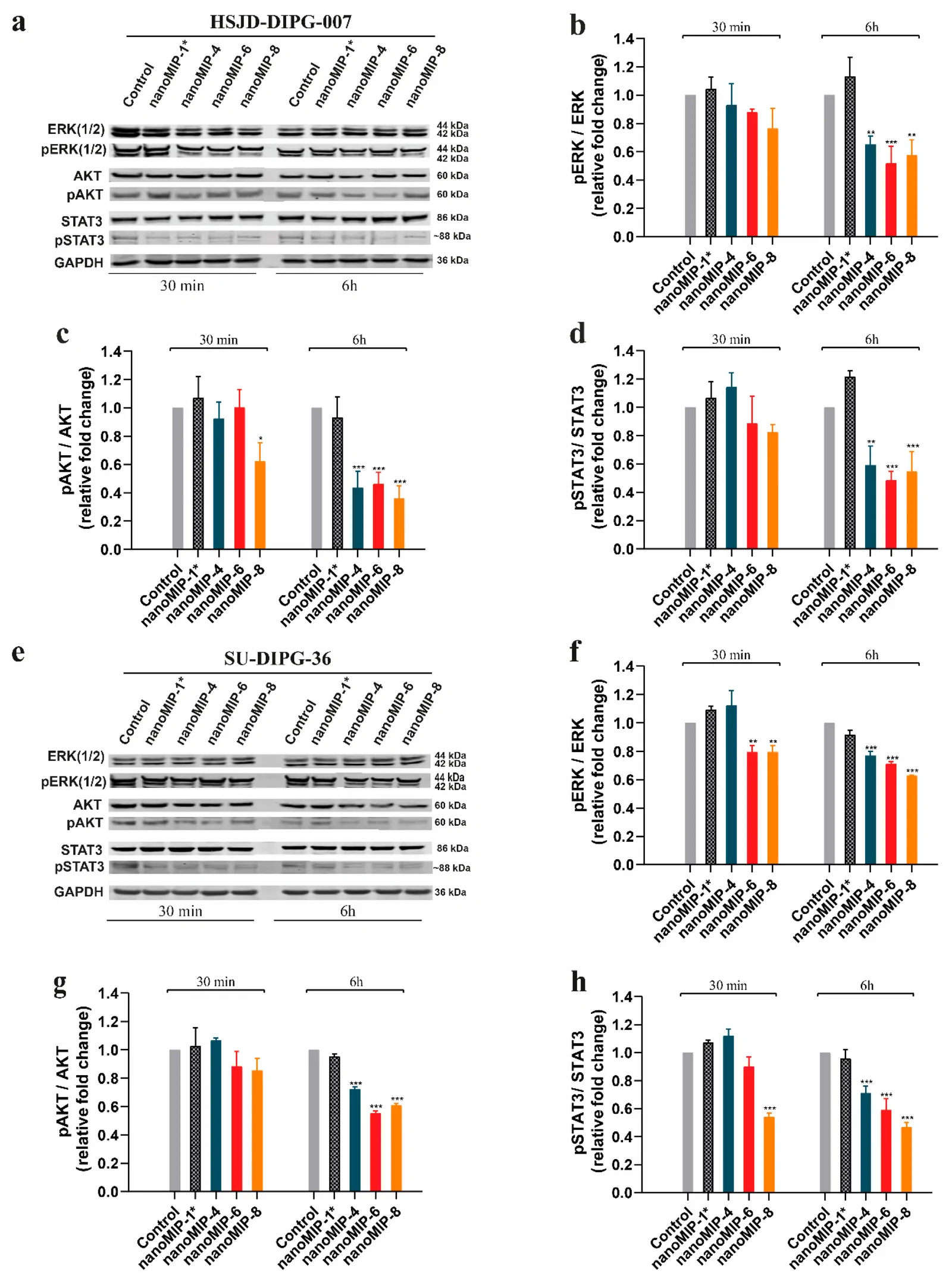
Western blot tracks of (**a**) HSJD-DIPG-007 cells, and quantified phosphorylation ratios for (**b**) p-ERK/ERK, (**c**) p-AKT/AKT, and (**d**) pSTAT3/STAT3 after nanoMIP treatment activation. (**e**) Western blot tracks of SU-DIPG-36 cells, and quantified phosphorylation ratios for (**f**) p-ERK/ERK, (**g**) p-AKT/AKT, and (**h**) pSTAT3/STAT3 after nanoMIP treatment activation (* *p* < 0.05, ** *p* < 0.005, *** *p* < 0.001).

To determine the molecular mechanism of nanoMIP driving the observed anti-proliferative effect, Western blot assays were conducted to analyze core intercellular survival pathways. HSJD-DIPG-007 and SU-DIPG-36 cells were analyzed after 30 min and 6 h of nanoMIP-4, nanoMIP-6, nanoMIP-8 or nanoMIP1* exposure, Representative Western blot images were summarized in Figure 4a–f and full-page Western blot images were given in Figures S6 and S7, respectively. While signal states remained unchanged at the brief 30 min checkpoints, treatment with nanoMIP-4, nanoMIP-6 or nanoMIP-8 for 6 h resulted in significant reductions in MAPK/ERK, PI3K/AKT, and STAT3 phosphorylation across both patient-derived glioma lines, indicating a clear shutdown of the mitogen-activated protein kinase (MAPK) signaling axis (Figure 4a,b).

Similarly, AKT phosphorylation at Ser473 was significantly suppressed after 6 h of treatment, indicating that these payload-free nanoMIPs inhibit the PI3K/AKT survival cascade (Figure 4a–c,e–g). Furthermore, activation of the STAT3 oncogenic transcription factor (monitored via Tyr705 phosphorylation) was heavily down-regulated (Figure 4a–h). Because STAT3 activation drives the expression of critical downstream anti-apoptotic genes, its rapid suppression suggests the initiation of programmed cell death pathways. In contrast, the control nanoMIP-1* produced no detectable alterations in pathway activation states, matching vehicle-treat baseline controls.

Because the extracellular portion of IL-13Rα2 lacks an autonomous intracellular signaling domain, it is highly likely that its oncogenic activity is mediated via structural crosstalk with a prominent signal-transducing co-receptor. In glioblastoma configurations, IL-13Rα2 physically pairs with the tumor-specific mutation receptor EGFRvIII [21] to form a functional signaling complex that constitutively drives the downstream PI3K/AKT, MAPK and STAT pathways [62]. By specifically binding the critical D1 and D3 outer binding domains of IL-13Rα2, our custom nanoMIPs block these viral receptor interactions, acting as functional antagonists to shut down intracellular survival signaling without requiring an internal toxic drug payload [63,64].

Our study showed that binding of IL-13Rα2-specific nanoMIPs to glioma cells correlated with decreased phosphorylation of ERK1/2, AKT, and STAT3, which are pro-survival pathways, and with reduced cell viability (Figure 2 and Figure 4). Since IL-13Rα2 lacks a cytoplasmic signaling domain [21], it is likely signaling through interaction with a cancer-specific form of EGFRvIII [62], which constitutively activates PI3/AKT, MAPK and STAT pathways in glioblastoma [65]. IL-13 can also bind IL-13Rα1 with low affinity when IL-13Rα1 heterodimerizes with IL-4Rα. This complex activates Tyk2, Jak2, Jak1 kinases, and STAT6, which are involved in apoptosis [66]. This also induces expression of 15-lipoxygenase-1 (ALOX15), an enzyme linked to apoptosis [63] and consequently PPARγ, a receptor known to suppress tumor growth [64]. Thus IL-13Rα2 appears to be twofold: (i) to prevent apoptosis and (ii) to promote glioma cell proliferation.

### 3.7. Effects of nanoMIPs on Caspase Activation in Glioma Cells

**Figure 5:**
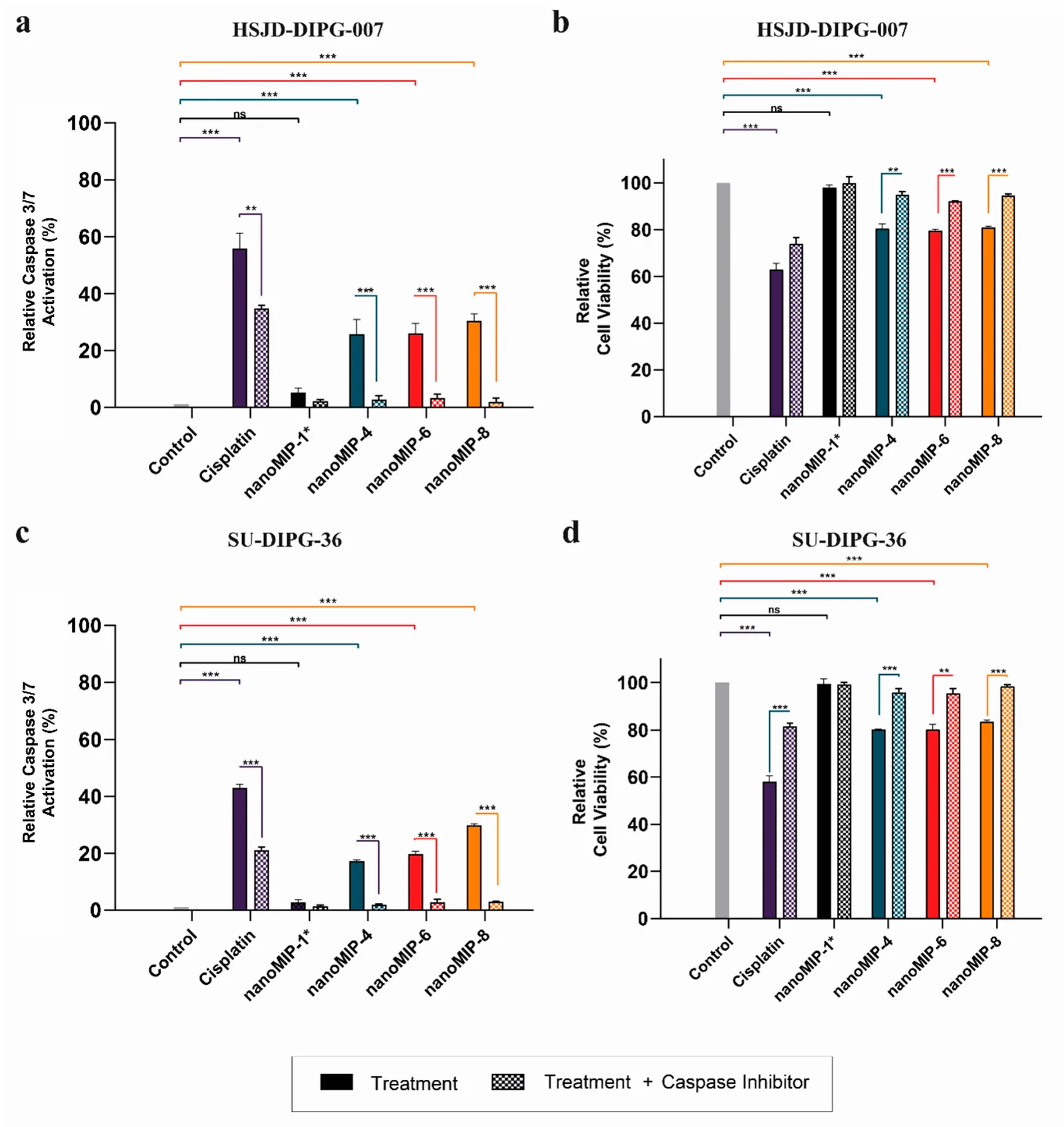
Quantified caspase-3/7 activation profiles and corresponding cell viability data using 100 ppm of nanoMIP4, nanoMIP-6 and nanoMIP-8, nanoMIP-1* (reverse sequence, negative control) and 25 µM of cisplatin (positive control). (**a**) Caspase-3/7 activation for 24 h treatment in HSJD-DIPG-007 cell. (**b**) Cell viability data with or without pan-caspase inhibitor in HSJD-DIPG-007 cell. (**c**) Caspase-3/7 activation for 24 h treatment in SU-DIPG-36 cells. (**d**) Cell viability data with or without pan-caspase inhibitor in SU-DIPG-36 (** *p* < 0.005, *** *p* < 0.001).

To determine if the loss of metabolic cell viability was directly caused by the induction of apoptosis, we monitored the activation profiles of executioner caspase-3 and 7 using the Caspase-Glo^®^ assay. Exposure to lead variants nanoMIP-4, nanoMIP-6 and nanoMIP-8 for 24 h caused a significant increase in active caspase-3/7 levels within both HSJF-DIPG-007 and SU-DIPG-36 cells, mirroring the trends observed in the cisplatin positive control group. The reserve-sequence control nanoMIP-1* caused no detectable change in caspase activation (Figure 5a,c).

To confirm that cell death was mediated via this enzymatic cascade, parallel cell cohorts were pre-treated for 2 h with the pan-caspase inhibitor Z-VAD-FMK (50 µM) prior to nanoMIP addition. The addition of Z-VAD-FMK significantly blocked the activation of caspase-3/7 across all nanoMIP treatment groups. Crucially, this caspase blockade successfully rescued the cells from polymer-induced cytotoxicity, returning viability levels toward baseline values (Figure 5b,d). Furthermore, we included a positive control cisplatin treatment group due to being a known inducer of apoptosis, which activates caspase-3/7 in glioma cells [67,68], providing a baseline for comparison with the effects of nanoMIPs on cell death pathways. These findings confirm that these engineered polymer nanoparticles selectively trigger a caspase-dependent apoptotic caspase inside patient-derived DIPG cells.

### 3.8. Binding Interface and Activity Relationship Between nanoMIPs and IL-13Rα2 Receptor

Structurally, the outer portion of IL-13Rα2 features a tree-domain layout: an N-terminal S-type Ig architecture (D1) followed by a pair of fibronectin III-like configurations (D2 and D3). Native binding mapping confirms that the receptor interacts with its natural ligand, IL-13, via a distinct bitopic mechanism where the ligand is physically grasped between the upper D1 and lower D3 regions [21]. IL-13Rα2 binds IL-13 through two key interfaces: site II and site III. In site II, the D2-D3 CHR loops of IL-13Rα2 accommodate into D helix residues of IL-13, stabilized by hydrogen bonds, salt bridges, and hydrophobic interactions. Site III features an extended β-sheet in the D1 Ig domain of IL-13Rα2, forming a flat, nonpolar interface with AB and CD loops of IL-13, reinforced by backbone hydrogen bonding and van der Waals forces. These interactions collectively stabilize the IL-13/IL-13Rα2 complex with high specificity [21,35].

Our screening results demonstrate that our most effective therapeutic formulations, nanoMIPs, nanoMIP-4, nanoMIP-6 and nanoMIP-8, map precisely onto these critical operational points: the D1 domain (residues: 113–127), and the D3 domain (residues: 329–338 and 275–286), respectively (Figure S4a,b). By blocking these regions with stable synthetic polymeric nanoMIPs, we disrupt the structural mechanics required for receptor complex coordination and downstream oncogenic signaling.

## 4. Conclusions

This study demonstrates that the targeted disruption of IL-13Rα2 signaling using rationally designed nanoMIPs represents a promising therapeutic approach for aggressive brain tumors. By using the EMMI method, we successfully determined target epitopes and engineered payload-free synthetic antibody nanoMIPs that bind specifically to critical extracellular domains (D1 and D3) of the IL-13Rα2 receptor. These nanoMIPs selectively target patient-derived DIPG cell lines overexpressing the receptor, while completely sparing non-tumoral glial cells. At the molecular level, this targeted interaction blocks crucial survival cascades, specifically inhibiting the phosphorylation of ERK1/2, AKT, and STAT3, and triggering a classic caspase-dependent apoptotic pathway without the need for a toxic chemotherapeutic payload.

Importantly, these nanoMIPs offer key translational advantages over classic biological monoclonal antibodies or short peptide systems, including high chemical and thermal stability, robust reproducibility, and the capacity to target small, sterically hindered functional epitopes. While this work establishes the foundation for in vitro efficacy and the mechanism of action of these specific formulations, it also provides a critical baseline for future further patient-derived cell and in vivo animal studies to evaluate blood–brain barrier penetration, biodistribution, and therapeutic safety.

## Data Availability

The datasets generated and/or analyzed during the current study are not publicity available due to institutional regulators but are available from the corresponding authors upon reasonable request.

## Supplementary Materials

The following supporting information can be downloaded at https://www.mdpi.com/article/10.3390/cancers18183028/s1, 2.3. LC-MS Sequencing protocol; Table S1. Epitope sequences of IL-13Rα2; Table S2. The hydrodynamic diameter and size distribution of the nanoMIPs; Figure S1. (a) IL13/ IL1-3Rα2 complex interaction domains and (b) nanoMIPs interaction domains; Figure S2. Synthesis of fluorescent labeled nanoMIPs by solid phase polymerization for selected epitopes sequences to target different motifs of IL-13Rα2 protein; Figure S3. Morphologies of selected nanoMIPs; Figure S4. Octet BLI association data obtained for 30 min association time for the constant concentration of each nanoMIPs and the binding kinetic constant (Kd) was determined for the selected nanoMIPs; Table S3. The mutations for the glioma cancer cell lines and the non-tumoral MO3.13 immortalized cell line; Figure S5. Cell viability profiles of MO3.13; Figure S6. Western blot tracks of HSJD-DIPG-007 cells after 30 min and 6h nanoMIP treatments activation; Figure S7. Western blot tracks of SU-DIPG-36 cells after 30 min 6h nanoMIP treatment activation.
