# Supplementary material for: Payload-Free Epitope Imprinted Polymer Nanoparticles Selectively Targeting IL-13Rα2 and Inhibition of Diffuse Midline Glioma Cells
Source: Cancers (Basel). 2026 Sep 17;18(18):3028. doi: 10.3390/cancers18183028 (PMC13605598; doi:10.3390/cancers18183028)
Supplement: Supplementary file 1 [file cancers-18-03028-s001.zip › cancers-4526583-supplementary.pdf]

## Supplementary Information

# Payload-Free Epitope Imprinted Polymer Nanoparticles Selectively Targeting IL-13Rα2 and Inhibition of Diffuse Midline Glioma Cells

Damla Ulker [1,2,3*], Adem Ozleyen [3,4], Dmitry Pshezhetskiy[5], Elena Piletska[3], Salvador Macip[6,7], Nick A Barlev[8] and Sergey Piletsky[3*]

[1]School of Mathematical and Physical Sciences, University of Sheffield, Sheffield S3 7HF, UK

[2]Faculty of Pharmacy, Department of Pharmaceutical Basic Sciences, Near East University, Near East Bvd, TRNC Mersin 10, TR-99138, Türkiye.

[3]School of Chemistry, University of Leicester, Leicester, LE1 7RH, United Kingdom.

[4]Health Institutes of Türkiye, Türkiye Biotechnology Institute, 06270, Ankara, Türkiye.

[5]Norwich Medical School, University of East Anglia, Norwich, United Kingdom.

[6]Mechanisms of Cancer and Aging Laboratory, Department of Molecular and Cell Biology, University of Leicester, Leicester, United Kingdom.

[7]Epi4health, Faculty of Health Sciences, Universitat Oberta de Catalunya, Barcelona, Spain.

[8]Department of Biomedical Sciences, School of Medicine, Nazarbayev University, Astana, 01000, Kazakhstan

**Table of Contexts**

2.3. LC-MS Sequencing protocol ……………………………………………………..…….…………………3
Table S1. Epitope sequences of IL-13Rα2………………………………………..…...………………..3
Table S2. The hydrodynamic diameter and size distribution of the nanoMIPs…………..……………….……4
Figure S1. (a) IL13/ IL1-3Rα2 complex interaction domains and (b)nanoMIPs interaction domains ..... 5
Figure S2. Synthesis of fluorescent labeled nanoMIPs by solid phase polymerization for selected epitopes sequences to target different motifs of IL-13Rα2 protein. ...................................................... 5
Figure S3. Morphologies of selected nanoMIPs.. .................................................................................. 6
Figure S4. Octet BLI association data obtained for 30 min association time for the constant concentration of each nanoMIPs and the binding kinetic constant (Kd) was determined for the selected nanoMIPs. ............................................................................................................................................ 6
Table S3. The mutations for the glioma cancer cell lines and the non-tumoral MO3.13 immortalized cell line……………………………………………………………………………………………………………7
Figure S5. Cell viability profiles of MO3.13 ……………………………………………………………………..7
Figure S6. Western blot tracks of HSJD-DIPG-007 cells after 30 min and 6h nanoMIP treatments activation …………………………………………………………………………………………………8
Figure S7. Western blot tracks of SU-DIPG-36 cells after 30 min 6h nanoMIP treatment activation.…..9

**Supporting Methods**

**2.3. LC-MS Sequencing Protocol**

Peptides were initially loaded onto a Waters 2G-V/M Symmetry C18 trap column (180 µm x 20 mm, 5 µm) for desalting and chromatographic focusing prior to elution onto a Waters Acquity HSS T3 analytical UPLC column (75 µm x 250 mm, 1.8 µm). The injection volume was 2 µL at a peptide concentration of 0.5 ug/µL. The inherent sensitivity of the instrument is 1 fMol on column. Single pump trapping was utilized with 99.9% solvent A and 0.1% solvent B at a flow rate of 5 µL/min for 3 min. Solvent A consisted of LC-MS grade water containing 0.1% formic acid, and solvent B was acetonitrile containing 0.1% formic acid. For the analytical column, the flow rate was set at 0.3 µL/min and the temperature was maintained at 40 °C. A 50 min run time gradient elution was initiated as the peptides eluted from the trap column using the following profile: 0 min, 3% B; 30 min, 40% B, 32 min, 85% B; 40 min, 85% B; and 41 min, 3% B. The NanoAcquity UPLC was coupled to a Waters Synapt G2 HDMS mass spectrometer operating in positive electrospray ionization mode. The capillary voltage was set at 2.4 kV and cone voltage at 30 V. PicoTip emitters (New Objective, US, 10 µm internal diameter) were used for the nano stage probe. A helium gas flow of 180 mL/min and an ion mobility separator nitrogen gas flow of 90 mL/min at 2.5 mbar were maintained. The IMS wave velocity was set at 650 m/s and the IMS wave height at 40 V. During HDMSE acquisition, a low collision, induced dissociation energy of 4 V was applied across the transfer ion guide, while a ramp of 20 to 40 V was applied for high collision energy acquisition. Argon was used as the collision induced dissociation gas. Lockspray mass accuracy was maintained throughout the chromatographic run using [Glu1]-Fibrinopeptide with m/z 785.8427. Data were acquired and analyzed using MassLynx 4.1.

**Supporting Results**

**Table S1.** Epitope sequences of IL-13Rα2

*Unique and used sequences present, but the whole list over 15000 sequences was not included

| | **Sequence** | **Retention time (min)** | **Neutral mass** | **Score** |
|---|---|---|---|---|
| 1 | IGCLYTFLI | 27.54858333 | 1330.676267 | 7.7029 |
| 2 | YTFLISTTFGC | 44.3171 | 2184.070633 | 7.6641 |
| 3 | TSSSDTEIKV | 25.54215 | 1684.758039 | 7.9544 |
| 4 | QPPLSLDHFKECTVEYE | 56.80213333 | 2908.430964 | 7.388 |
| 5 | YLQWQPPLSLDHFK | 53.22863333 | 1770.918985 | 8.6664 |
| 6 | QPPLSLDHFKECTVEYE | 54.24875 | 2033.977 | 6.8642 |
| 7 | YTFLISTTFGC | 44.3171 | 2184.070633 | 7.6641 |
| 8 | NIGSETWKTIITK | 42.36119167 | 1603.887917 | 8.2678 |
| 9 | GSETWKTIIT | 42.76998333 | 1262.704659 | 9.2984 |
| 10 | IITKNLHYKDGF | 49.28313 | 1447.823 | 6.3294 |
| 11 | FPYLEASDYK | 43.72248333 | 1231.60273 | 9.1333 |

| | | | | |
|---|---|---|---|---|
| 12 | QCWEGEDLSK | 40.0333 | 1663.835152 | 7.6794 |
| 13 | EWSDKQCWEGEDLSKKTLL | 47.31053333 | 2294.16618 | 7.4693 |
| 14 | DLNKGI | 25.15028 | 658.385 | 7.3988 |
| 15 | AKIHTLLPWQCTNGS | 33.95995 | 2023.992 | 6.5481 |
| 16 | CTNGSEVQSSWAET | 83.50147 | 2543.145 | 6.7254 |
| 17 | SEVQSSWAETTYWI | 40.20237 | 1685.793 | 7.286 |
| 18 | GSEVQSSWAETTY | 41.01985 | 2244.931 | 6.9736 |
| 19 | AETTYWISPQGIPETKVQD | 44.30019167 | 2162.088131 | 7.9716 |
| 20 | QGIPETKVQDMDCV | 41.35797 | 1561.741 | 7.2396 |
| 21 | YNWQYLLC | 28.00792 | 1101.467 | 7.4736 |
| 22 | LLCSWKPGIGVL | 43.4322 | 1284.674 | 6.7754 |
| 23 | WKPGIGVLLDTNYNLF | 25.57597 | 1848.962 | 6.6229 |
| 24 | LFYWYEGLDH | 41.01985 | 1455.662 | 7.2165 |
| 25 | GLDHALQCVDYIKAD | 53.7556 | 2561.23 | 6.8701 |
| 26 | ICVNGSSENKPIR | 34.25021667 | 1502.79702 | 7.6463 |
| 27 | LPPVYLTFTRESSC | 41.35796667 | 1708.817855 | 7.4522 |
| 28 | LPPVYLTFTRESSCEIK | 46.61407 | 1982.064 | 7.0291 |
| 29 | SIPLGPIPARCFDYE | 48.16158333 | 1789.90289 | 7.3066 |
| 30 | SIPLGPIPARCFDYEIEI | 26.52847 | 2032.053 | 6.6194 |
| 31 | YEIEIREDDTTL | 36.78395 | 1495.735574 | 7.7724 |
| 32 | YEIEIREDDTTLVTATVENETY | 54.43758333 | 2603.186373 | 7.7755 |
| 33 | IEIREDDTTLVTATVEN | 42.88833333 | 1917.926961 | 7.5166 |
| 34 | EDDTTLVTAT | 34.74338333 | 1064.500834 | 7.5835 |
| 35 | YTLKTTNETR | 23.637 | 1326.695 | 7.0626 |
| 36 | TTNETRQLCFVVRS | 53.72178333 | 1652.841146 | 7.5463 |
| 37 | IPARCFDYEIEIREDDTTLVTA | 50.69511667 | 2569.268914 | 6.9522 |
| 38 | KPNTYPKMIPEFFC | 27.57112778 | 1713.804597 | 7.4846 |
| 39 | WSIPLGPIPAR | 53.72178333 | 1205.713974 | 8.983 |
| 40 | PLGPIPAR | 53.72178333 | 819.5086134 | 8.5365 |

**Table S2.** The hydrodynamic diameter and size distribution of the nanoMIPs
**The measurements were conducted using dynamic light scattering (DLS) in aqueous media at 25 °C, as well as after overnight incubation in PBS and TMS media at 37 °C.*

| nanoMIPs code | (d.nm)/PDI aqueous media at 25 $^{o}$C | (d.nm)/PDI in PBS at 37 $^{o}$C* | (d.nm)/PDI in TMS at 37 $^{o}$C* |
|---|---|---|---|
| nanoMIP-1 | 147.0/ 0.34 | 129.9/ 0.32 | 121.8/ 0.20 |
| nanoMIP-1* | 158.4/ 0.31 | 154.0/ 0.24 | 145.7/ 0.18 |
| nanoMIP-2 | 146.1/ 0.25 | 161.5/ 0.27 | 139.1/ 0.21 |
| nanoMIP-3 | 113.4/ 0.25 | 124.6/ 0.21 | 129.4/ 0.25 |
| nanoMIP-4 | 154.1/ 0.20 | 167.9/ 0.20 | 164.1/ 0.15 |
| nanoMIP-5 | 144.1/ 0.20 | 148.3/ 0.18 | 141.9/ 0.26 |
| nanoMIP-6 | 152.5/ 0.21 | 158.7/ 0.07 | 149.5/ 0.13 |
| nanoMIP-7 | 154.1/ 0.07 | 172.6/ 0.04 | 169.5/ 0.32 |
| nanoMIP-8 | 153.0/ 0.34 | 160.2/ 0.20 | 145.7/ 0.23 |
| nanoMIP-9 | 163.2/ 0.20 | 170.4/ 0.21 | 174.1/ 0.18 |

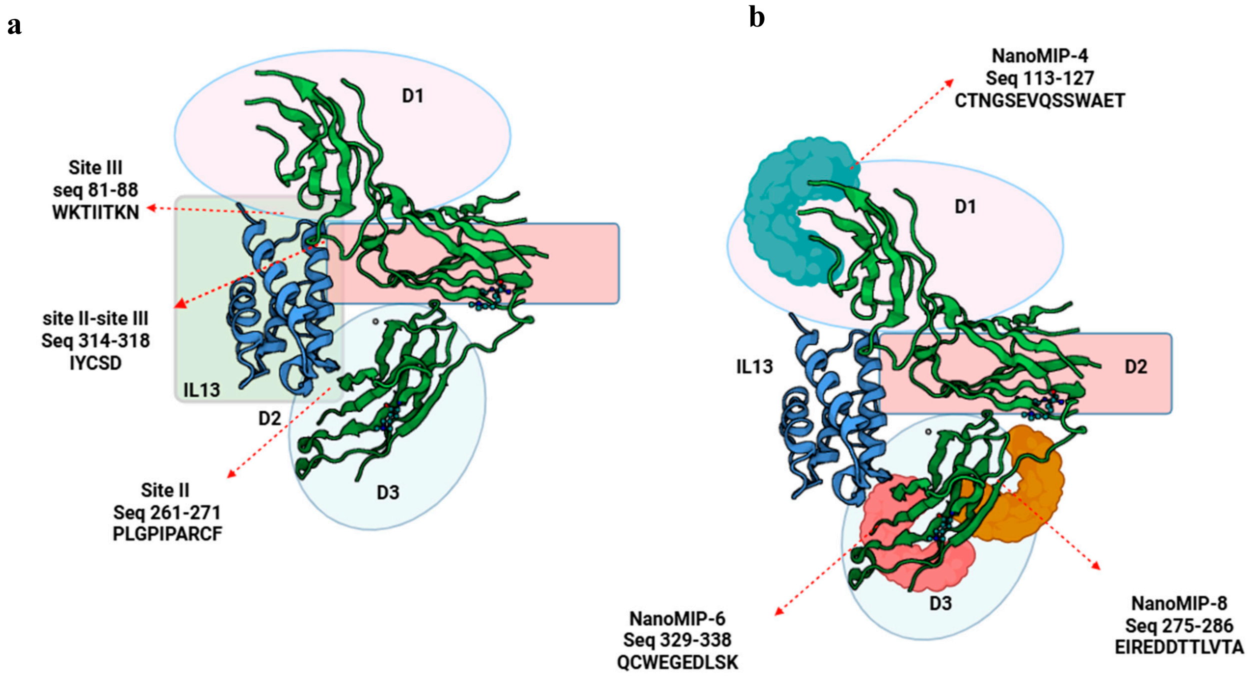


**Figure S1.** (a) IL13/ IL1-3Rα2 complex interaction domains and (b)nanoMIPs interaction domains. This image is created with BioRender.com

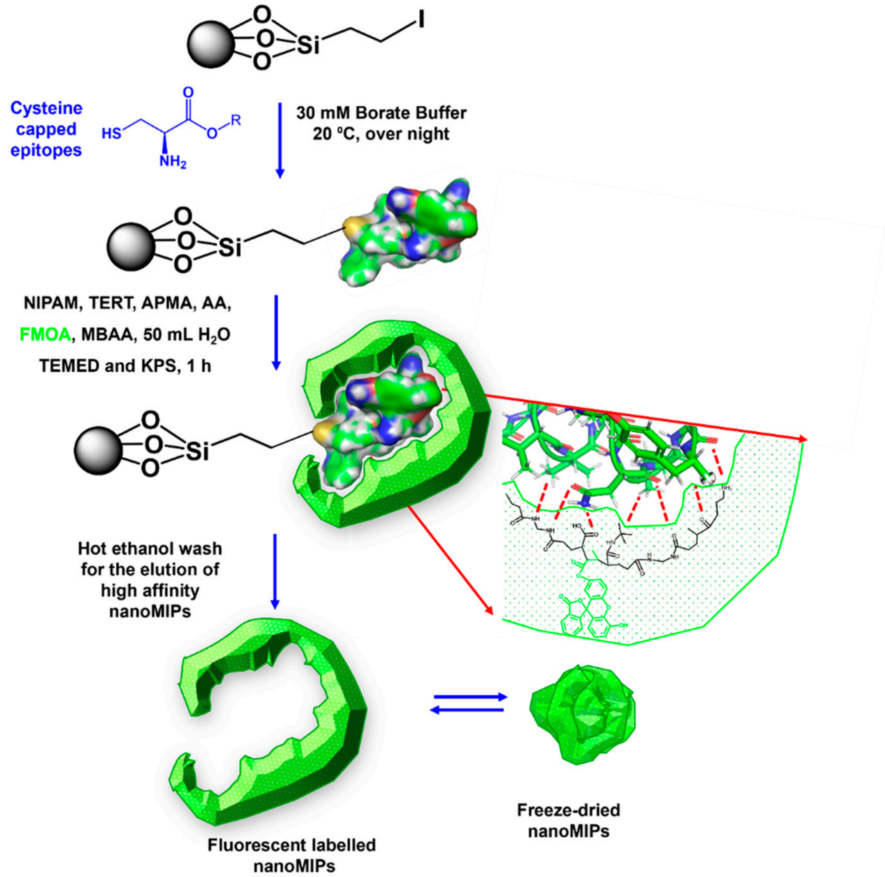


**Figure S2.** Synthesis of fluorescent labeled nanoMIPs by solid phase polymerization for selected epitope sequences to target different motifs of IL-13Rα2 protein.

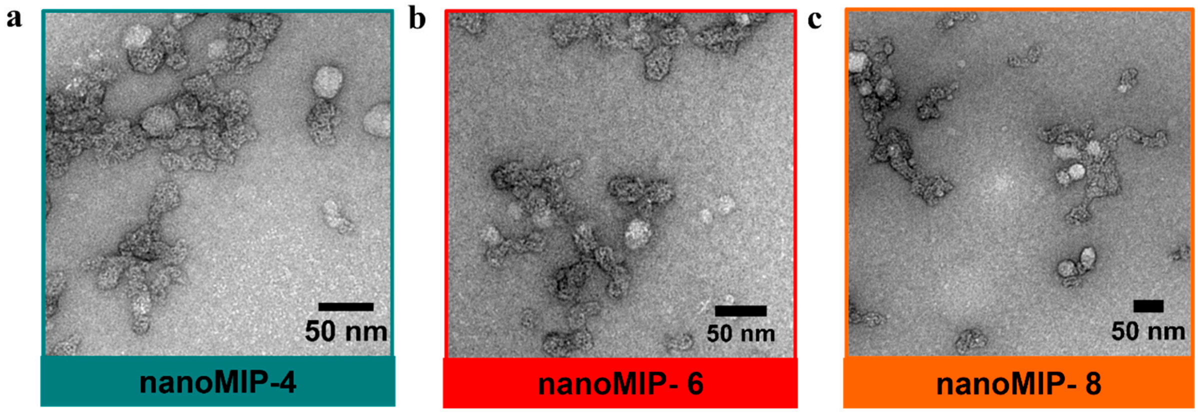


**Figure S3.** Morphologies of selected nanoMIPs. The samples were prepared at 0.03% (w/v) in aqueous media, and the representative TEM images of summarized a: nanoMIP-4, b: nanoMIP-6 and c: nanoMIP-8 were recorded after drying at 25 °C.

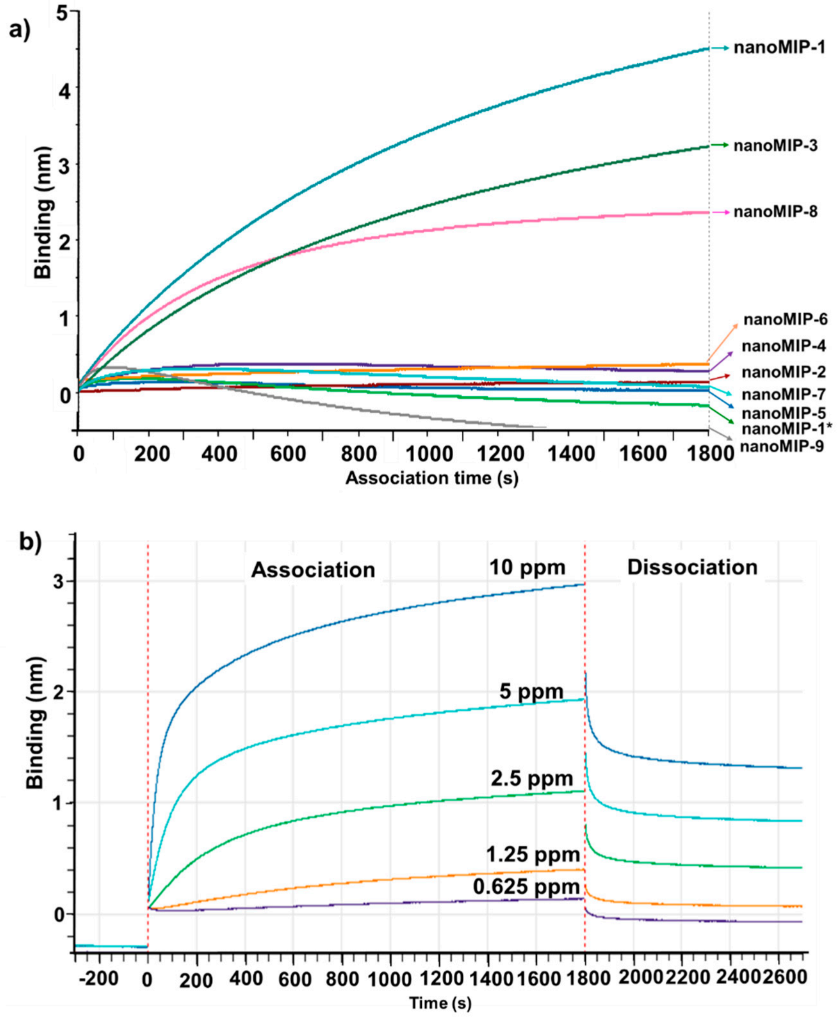


**Figure S4.** Octet BLI association data obtained for 30 min association time for the constant concentration of each nanoMIPs and the binding kinetic constant (Kd) was determined for the selected nanoMIPs; a) a concentration of 100 ppm of nanoMIP-1, nanoMIP-1*, nanoMIP-2, nanoMIP-3, nanoMIP-4, nanoMIP-5, nanoMIP-6, nanoMIP-7, nanoMIP-8, and nanoMIP-9 was immobilized on the biosensor surface. The association study was then conducted to compare data using 10 ppm of IL13Rα2 in 10 mM acetate buffer (pH 4) at 30 $^{o}$C, b) the representative graph of 100 ppm of nanoMIPs-1 towards 10 ppm, 5 ppm, 2.5 ppm, 1,25 ppm and 0,0625 ppm IL13Rα2 in 10 mM acetate buffer (pH 4) at 30 *$^{o}$C.*

**Table S3.** The mutations for the glioma cancer cell lines and the non-tumoral MO3.13 immortalized cell line.

| Cell line | Mutations |
|---|---|
| HSJD-DIPG-007 | H3.3K27M, ACVR1, PIK3CA, MYC (amp), PPM1D |
| SU-DIPG-36 | H3.3K27M, ACVR1 |
| MO3.13 | Not applicable mutation to H3.3K27M |

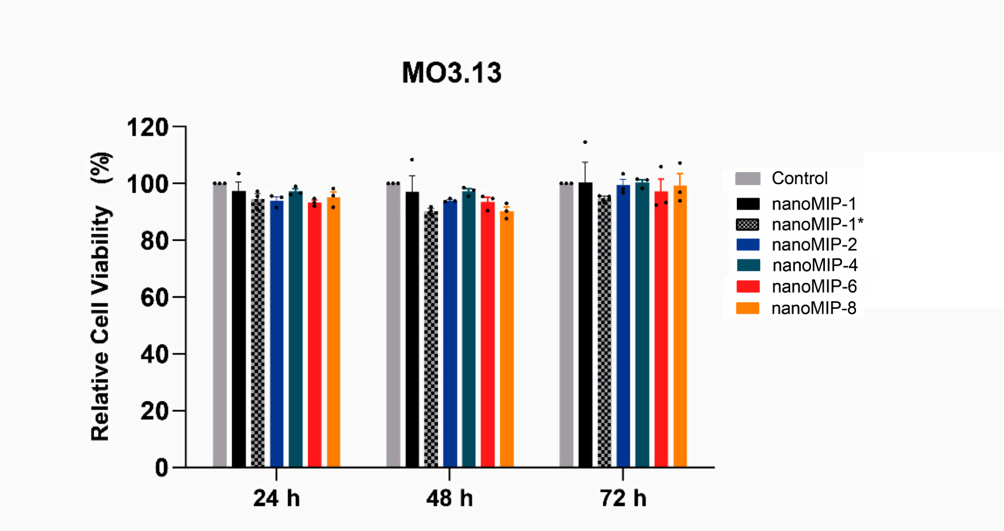


**Figure S5.** Cell viability profiles of MO3.13 cells treated with the nanoMIP-1, nanoMIP-1*, nanoMIP-2, nanoMIP-4, nanoMIP-6 and nanoMIP-8 over 24, 48 and 72 hours. The data were obtained from at least three technical and/or biological replicates using a Hidex Sense plate reader (LabLogic, UK) to determine the luminescence measurements and are presented as the mean ± standard error of the mean (SEM). Statistical significance between control (C: DMEM cell media control) and treatment groups was assessed using one-way analysis of variance (ANOVA) followed by Dunnett's post hoc test, performed with GraphPad Prism 9 software (*$p < 0.05$, **$p < 0.005$, ***$p < 0.001$).

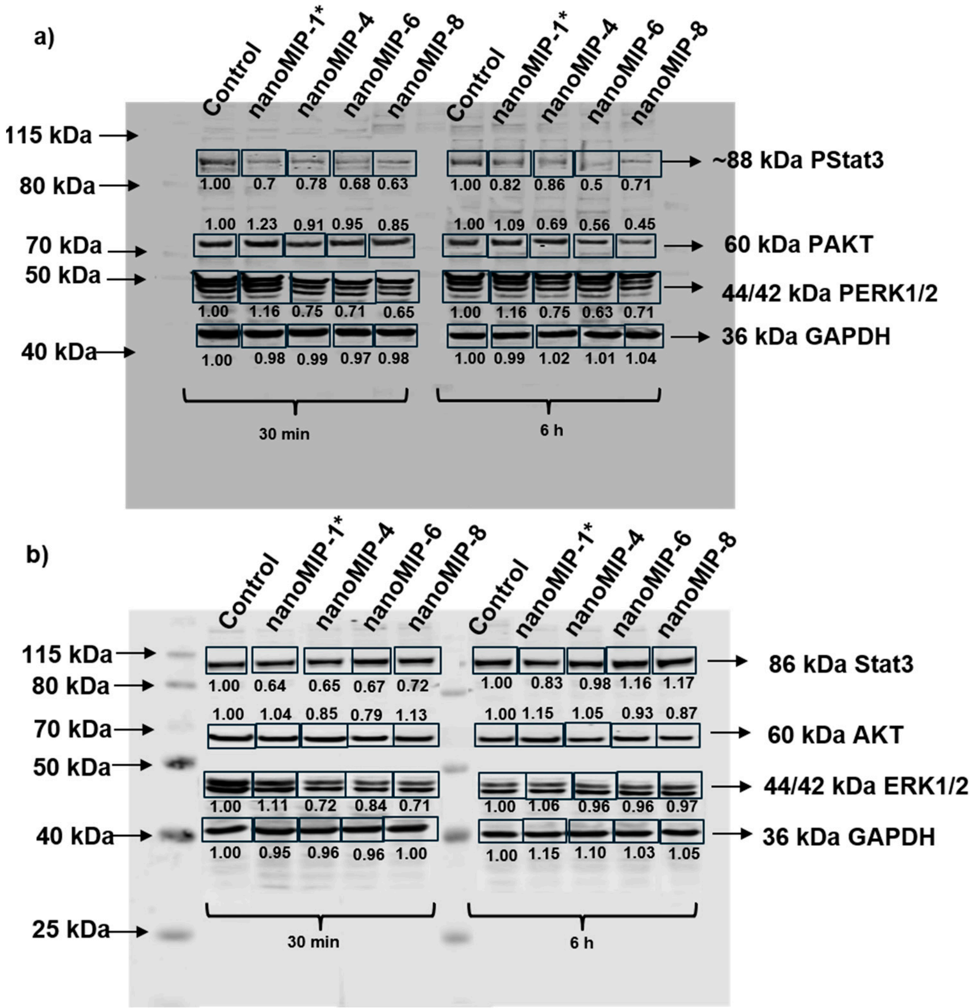


**Figure S6.** Western blot tracks of HSJD-DIPG-007 cells after 30 min and 6h nanoMIP treatments activation.

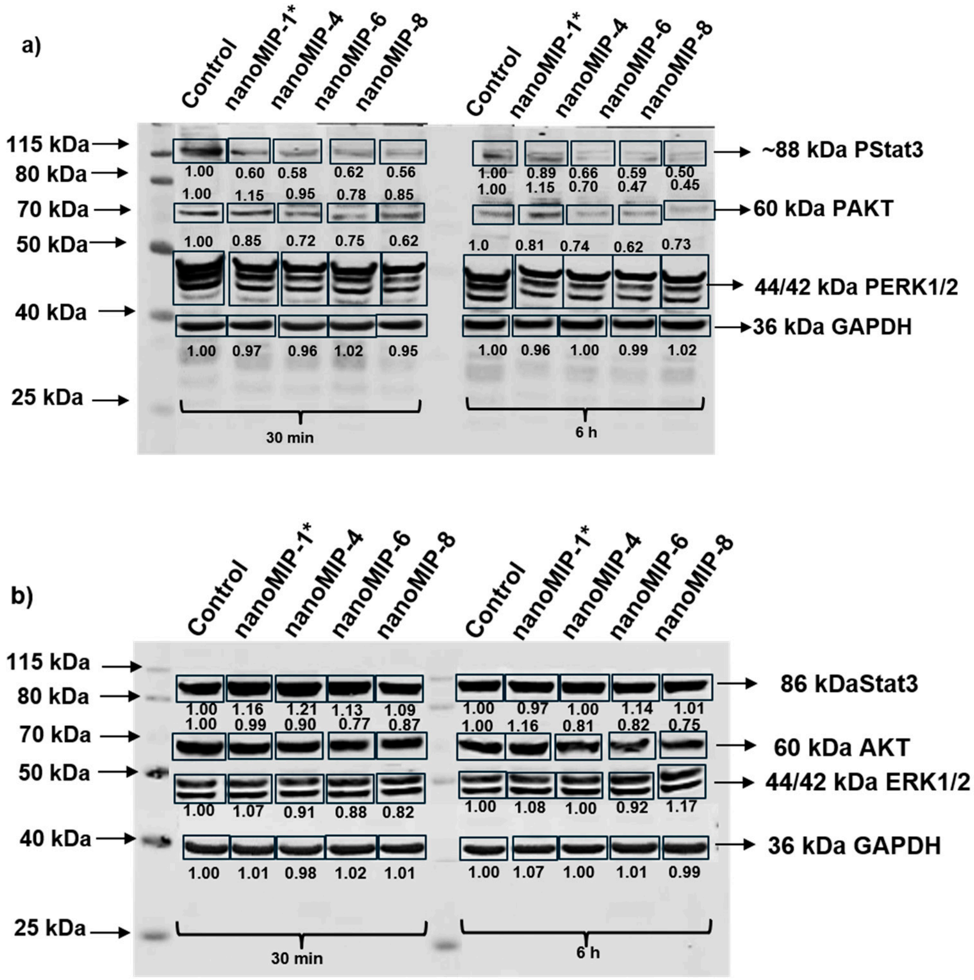


**Figure S7.** Western blot tracks of SU-DIPG-36 cells after 30 min 6h nanoMIP treatment activation.
